# TKA following high tibial osteotomy versus primary TKA - a matched pair analysis

**DOI:** 10.1186/1471-2474-11-207

**Published:** 2010-09-14

**Authors:** Turgay Efe, Thomas J Heyse, Christoph Boese, Nina Timmesfeld, Susanne Fuchs-Winkelmann, Jan Schmitt, Christina Theisen, Markus D Schofer

**Affiliations:** 1 Department of Orthopaedics and Rheumatology, University Hospital Marburg, Baldingerstrasse, 35043 Marburg, Germany; 2 Institute of Medical Biometry and Epidemiology, Philipps-University Marburg, Bunsenstrasse 3, 35037 Marburg, Germany

## Abstract

**Background:**

High tibial osteotomy (HTO) is a well established technique for the treatment of medial osteoarthritis of the knee with varus malalignment. Results of total knee arthroplasty (TKA) after previous HTO are still discussed controversially. The aim of this study was to elucidate the clinical and radiological results as well as perioperative data of prior HTO on TKA.

**Methods:**

Forty-one TKA after HTO were compared to 41 primary TKA at minimum of six years follow-up. Patients were matched according to age, gender, follow-up, etiology, and prosthetic design. Surgical data and complications were evaluated. Clinical outcome was assessed using a number of clinical scores and the visual analogue scale (VAS) for pain. X-rays were evaluated by the method of the American Knee Society. The patellar position was measured by the Insall-Salvati ratio.

**Results:**

There was no significant difference in mean operation time (p = 0.47) and complication rate (p = 0.08). The Knee Score of the KSS (p = 0.0007) and the ROM (p = 0.006 for extension and p = 0.004 for flexion, respectively) were significantly better in the control group. Mid-term results of the VAS, WOMAC, Lequesne, UCLA, Feller's Patellar Score and SF-36 showed no significant difference. Femoral and tibial component alignment were similar in both groups. One tibial component showed suspect radiolucencies in the HTO group. The Insall-Salvati ratio showed three patients with patella alta and one patient with patella baja in the HTO group. At latest follow-up all implants were still in place.

**Conclusions:**

Evaluating the clinical and radiological outcome, significant differences were only detected for range of motion and the Knee Score of the KSS. The present study suggests that the results of TKA with and without prior HTO are mainly identical. Although patients with a previous HTO had more complications, no statistically significant differences were noted with this group size.

## Background

According to the Swedish arthroplasty register [1] the number of surgical interventions due to osteoarthritis (OA) of the knee in younger patients (< 55 years) has doubled in the last decade. High tibial osteotomy (HTO) is a proven treatment option for osteoarthritis of the medial compartment. Although the short-term follow-up success of tibial osteotomy has shown good clinical outcome, the results seem to gradually deteriorate over time [2-4]. Corresponding to the demographic transition towards a higher average age in our society, more patients may require total knee arthroplasty (TKA) after failed osteotomy. Primary TKA is a popular and well established method for treatment of advanced degenerative joint disease with survival rates of 90 to 95% at 10 to 15 years [5-7]. There is evidence that TKA after previous HTO is technically more difficult and implies greater risk of complications than primary TKA [8,9]. Great debate whether a previously performed HTO may influence the clinical and radiological outcome of later TKA is still on-going. Several authors reported that previous HTO makes minimal or no difference [10-12] in the outcome of TKA while others showed poorer results [9,13,14].

The aim of the present study was twofold. In the first part of the study surgical data and clinical outcome of TKA after a previous HTO were compared with a cohort of patients who had undergone a primary TKA. In the second part of the study the radiological outcome of TKA after a previous HTO was compared with the control group.

## Methods

In a retrospective approach, 1519 cases of TKA performed between 1998 and 2003 at the authors' institution were identified from medical records. Among these cases there were 41 patients requiring surgery after previous HTO. Groups were matched according to age, gender, follow-up, etiology and prosthetic design. According to Rand et al. [15] there is a hazard ratio for each of the risk factors gender, age, etiology, prosthesis design and follow-up. Using the log hazard ratio, the beta-value can be recalculated. By adding the beta values of the different risk factors for each patient, an individual risk score is obtained. Subsequently, partners within the groups of minimal follow-up time differences with nearly identical risk factors were matched. Allocation of matched pairs was performed within an interval of +/-3 months of follow-up time. By this method the risk factors are compared in the overall context according to their relevance expressed by their hazard ratio. The study was authorised by the local ethical committee (No. 95/07).

The HTO group included 21 male and 20 female patients and the control group 17 male and 24 female patients. The average age at tibial osteotomy was 53 ± 4 (42-60) years. The mean time interval between HTO and TKA was 86 ± 19 (60-130) months. The average age at the time of examination was 69 ± 8 (51-84) years in the HTO group and 73 ± 7 (55-85) years in the control group. Follow-up examination following TKA was performed after an average of 82 ± 22 (48-121) months in the HTO group and after an average of 85 ± 20 (50-121) months in the control group.

The indication for HTO was symptomatic medial compartment osteoarthritis with varus malalignment. The conversion from HTO to TKA included radiographic progression of the osteoarthritis and increasing pain. In the HTO and control group primary degenerative osteoarthritis was seen in 40 patients, and rheumatoid arthritis in 1 patient. It is accepted that HTO is contraindicated in subjects with inflammatory arthritis but the diagnosis had been detected after the osteotomy. HTO was performed in 22 cases on the right and 19 on the left side. 20 primary TKA were performed on the right and 21 on the left side. All patients were operated on using the lateral closing-wedge technique as popularised by Coventry [16]. Fibular transection was performed at the junction of the middle and distal thirds [17], through a separate incision. A transverse incision with the patient in supine position was performed for the tibial osteotomy. Peroneal nerve was exposed and protected. The osteotomy was performed below the tibial tuberosity leaving the medial cortex intact. The bone wedge size was based on the preoperative calculations from the long leg standing radiograph. A laterally-based wedge of bone was removed and the osteotomy was fixed with an AO-plate.

**Figure 1:**
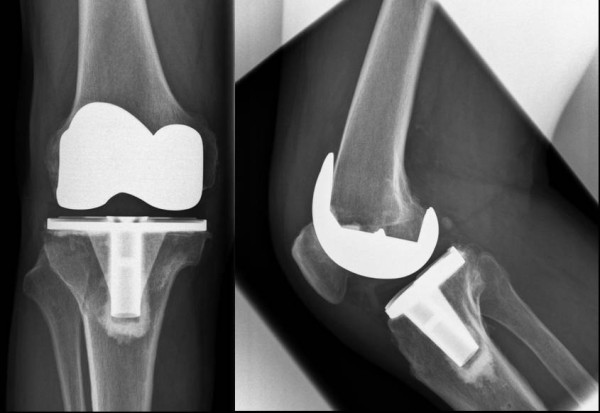
**Implantation of a NexGen LPS TKA after previous closed-wedge osteotomy**. The roentgenograms at the latest follow-up show good alignment and patella baja.

In all cases hardware removal through the same incision was performed after 1 year when bony consolidation of the osteotomy was completed. The indication for primary TKA occurred due to a considerable increase of osteoarthritis and pain. A cemented posterior-stabilised prosthesis (NexGen LPS, Zimmer, Warsaw, IN, USA) was implanted in all cases (Fig. 1). After a midline skin incision, the standard medial parapatellar capsular approach was used in all cases. Patellar resurfacing was not undertaken.

The clinical results were evaluated by the Western Ontario and McMaster Universities (WOMAC) Osteoarthritis Index [18], Visual Analog Scale (VAS), Knee Society Score (KSS) [19], Lequesne Index [20] and University of California Los Angeles Activity Assessment (UCLA) [21], as well as Feller's Patellar Score [22] and Medical Outcomes Study Short-Form 36 (SF-36) Health Survey [23] at the final follow-up. The ROM of the knee was measured using a goniometer. Standardised conventional X-rays in antero-posterior, lateral and skyline view of the patella in 30° flexion were taken. The radiological results were examined by the radiographic evaluation method of the American Knee Society [24]. Thereby the positioning of the prosthetic components was evaluated by measuring specific angles (α, β, γ, δ) in relation to the anatomical femoro-tibial axes and radiolucent lines. The femoral and tibial components were divided into seven zones in the antero-posterior and lateral view, the tibial component into three zones in the lateral view. The numerical score for the components was determined by measuring the width of the radiolucent lines for each of the zones in millimetres. For a seven-zone component, a score of 4 or less is regarded as stable, a score between 5 and 9 should be monitored for possible progression, and 10 or more indicates loosening. Patellar height was measured according to Insall-Salvati (IS) ratio [25]. All patients were investigated by a physical examination and the application of clinical scores by co-author CB, an orthopaedic resident. An independent specialist in radiology evaluated the diagnostic images. All of the osteotomies and arthroplasties were performed by two surgeons. The operating surgeons were not involved in the clinical assessment.

### Statistical analysis

The outcome of TKA after HTO was compared with primary TKA using the Welch's two-sample t-test. The comparison of the complication rate was performed with the Fisher's exact test. The level of significance was defined as P < 0.05.

## Results

The mean operation time showed no statistically significant difference (p = 0.47) between the HTO group with 95 ± 14 (75-115) minutes and the control group with 90 ± 11 (65-105) minutes. The HTO group required three lateral releases, one medial tightening and four synovectomies compared to four synovectomies in the control group. Eight complications (19.5%) were noticed in the HTO group: four cases of skin necrosis at the proximal part of the tibia required revision, two stiff knees (flexion < 90°) required mobilisation under general anaesthesia, one superficial infection could be managed with antibiotics and there was one clinically apparent venous thrombosis. In the control group, two (4.8%) superficial infections responded to antibiotics. No significant difference was detected between the two groups with respect to complications (p = 0.08). Neurovascular injuries, delayed union and non-union, compartment syndrome and intra-articular fractures associated with HTO did not occur. There were no cases of deep infections in both groups.

Patients who had undergone a previous osteotomy showed less ROM. The mean extension and mean flexion was 1.7° ± 3.1° (0°-10°) and 106° ± 14.1 (55°-125°) in the HTO group, and 0.2° ± 1.1° (0°-5°) and 115° ± 13.2 (90°-140°) in the control group, respectively. These differences were statistically significant (p = 0.006 and p = 0.004, respectively). The mean VAS score in both groups was 1.2 (p = 0.96). The results of the SF-36, Feller, Lequesne, KSS, UCLA and WOMAC scores are shown in Table 1. Except the worse knee score of the KSS in the HTO group (p = 0.0007), the overall clinical outcome showed no significant difference with a trend in favour of the control group.

**Table 1:** Clinical scores

Scores		HTO group	Control group	P-value
WOMAC (0-100) higher scores indicates difficulties	Pain	14.8 ± 19.4 (0-70)	16.4 ± 24.1 (0-86)	0.74
	Stiffness	15.2 ± 17.2 (0-60)	21.1 ± 24.7 (0-90)	0.22
	Function	18 ± 18.2 (0-61)	21.3 ± 23.7 (0-84))	0.49

KSS (0-100) 0 poor- 100 excellent	Knee	78.8 ± 18.9 (37-100)	91 ± 11.0 (54-100)	**0.0007**
	Function	78.2 ± 21.1 (25-100)	87.8 ± 48.3 (35-100)	0.25

Lequesne (0-8) 1 mild, > 14 severe discomfort	Pain or discomfort	2 ± 1.8 (0-6)	2.1 ± 1.8 (0-8)	0.61
	Maximum distance walked	1.4 ± 1 (0-5)	1.4 ± 1 (0-5)	0.21
	Activities of daily living	2 ± 1.7 (0-7)	2.4 ± 2.3 (0-8)	0.21

UCLA (1-10) 1 low-10 high activity		5.7 ± 1.2 (2-7)	5..3 ± 1.4 (2-7)	0.16

Feller's Patellar Score (3-30) 3 poor- 30 excellent		25.9 ± 6.2 (6-30)	26.5 ±4.8 (11-30)	0.61

SF-36 (0-100) 0 poor-100 good health	Physical functioning	64.3 ± 28.9 (0-100)	56.1 ±3 1 (10-100)	0.22
	Role-physical	59.8 ± 44.7 (0-100)	58.5 ± 45.6 (0-100)	0.90
	Pain index	59.9 ± 20.4 (12-100)	52.4 ± 23.1 (20-82)	0.12
	General health perception	67.1 ± 21.7 (10-107)	69.3 ± 24.1 (30-107)	0.66
	Vitality	58.7 ± 20.2 (0-95)	52.6 ± 26.4 (10-100)	0.24
	Social functioning	63.4 ± 20.8 (0-875)	58.5 ± 18.4 (25-87)	0.26
	Role-emotional	77.2 ± 37.6 (0-100)	69.1 ± 43.7 (0-100)	0..37
	Mental health index	75.1 ± 18.3 (0-96)	67.7 ± 24.1 (8-100)	0.12

The values are listed as mean, with standard deviation and range. Significant difference is given in bold.

The radiographic assessments of both groups are given in Table 2. No significant difference for femoral and tibial component positioning was noticed between the two groups. In the HTO group 10 femoral and 5 tibial components showed small non-progressive radioluncencies, whereas in the control group 6 femoral and 5 tibial components had small non-progressive radioluncencies. A significant difference in radiolucencies could not be detected between the groups. Suspect radiolucencies were only present in one tibial component (5 mm) in the HTO group but the patient had no clinical symptoms. No suspect radiolucencies were seen in the control group. No component had to be revised. In the HTO group the mean IS ratio was 0.94 ± 0.17 (0.85-1.3) mm, and patients in the control group showed a mean IS ratio of 0.90 ± 0.15 (0.8-1.2) mm (p = 0.34). There were more outliers in the HTO group with three cases of patella alta and one case of patella baja.

**Table 2:** Radiographic assessment according to the Knee Society Roentgenographic Evaluation System

	HTO group	Control group	P-value
Femoral component flexion a-p (α)	97° ± 3.1° (92°-102°)	97° ± 3.2° (91°-104°)	0.76
Tibial component angle a-p (β)	87° ± 1.7° (82°-92°)	87° ± 2.1° (81°-93°)	0.86
Femoral component flexion lat (γ)	4.7° ± 2.7° (0°-10°)	5° ± 2.3° (0°-9°)	0.69
Tibial component angle lat (δ)	84° ± 1.4° (77°-90°)	82° ± 1° (73°-93°)	0.15
Loosening of the components			
Femoral lat (mm)	n = 4 (1); 3 (2); 1 (3); 2 (4)	n = 2 (1); 4 (2)	0.50
Tibial a-p and lat (mm)	n = 4 (1); 1 (4); 1 (5)	n = 2 (1); 3 (2)	0.17

The values of the specific angles are listed as mean, with standard deviation and range. Suspicious radiolucencies (5 mm) were noted in one tibial component in the HTO group. a-p: anteroposterior view, lat: lateral view.

## Discussion

The most important finding of the present study is that the outcome of primary TKA and TKA following HTO as recorded by clinical and radiological results were quite similar. Even though patients with previous HTO had more postoperative complications the difference was not statistically significant.

The present study has some limitations mainly due to its retrospective design. However, matched-pair analyses allow ruling out some bias by matching some of the contributing factors. Of course matching is only possible to a certain extent, limited by patient numbers and availability of data. Other factors that might have influenced the outcome but were not matched are e.g. the ASA classification and obesity. Of major interest is usually the revision rate after joint arthroplasty. Revision rates can not easily be assessed by a matched-pair study. Even though there is sufficient number of studies available on TKA outcomes after HTO, the results in literature show a great variability. Differences in outcome may be caused by wide heterogeneity among the studies and pooling of the results are a challenge, as described by van Raaij et al. [26] in a systematic review. In order to minimise the effect of variables such as age, gender, follow-up, etiology and prosthetic design we compared the results of 41 primary TKA with 41 TKA following closed-wedge HTO in a matched-pair analysis.

HTO is an accepted treatment of varus OA of the knee in active patients. There is some evidence that malalignment induces OA progression and even development. Perhaps correction of malaligment may have a positive effect on OA. Not all HTO patients will require TKA and survival of HTO after 10 years with TKA as an endpoint ranges between 51 and 92% among several studies [3,27-29]. However, performing TKA secondary to HTO may be related to difficulties. In the present study several interventions had to be done to align the patella in the HTO group. This did not prolong TKA significantly. The small difference in operation time is in contrast to other studies [14,30,31] with more technical difficulties in exposure of the proximal part of the tibia, altered anatomy and soft tissue imbalance. The complication rate in the HTO group was considerably higher than described by Cameron and Park [32] and Madan et al. [14] with 11.2% and 9.7%, respectively. This may be related to the various risk factors of the patients.

Even though almost all clinical scores showed no significant differences between both groups, there seems to be trend in favour of the control group. In contrast to other studies [11,12,33-35] there were significant differences in Part A of the KSS in disfavour of the HTO group. Probably this can be explained by the poorer range of motion. A systematic review by van Raaij et al. [26] showed less range of motion with a median of 10° in patients receiving TKA following HTO compared to patients with primary TKA. However, Miner et al. [36] revealed that for assessment of TKA outcome ROM is much less important than the overall results.

Mode of failure in TKA includes osteolysis, malaligment, or malpositioning [37]. In the present study suspect radiolucencies were only present in one tibial component. Haslam et al. [38] could demonstrate that most TKA failures tend to occur after 6 years or more. The mean follow-up of the present study was slightly longer than 7 years, a conclusion about loosening is therefore only possible to a certain extent. As observed by several authors [35,39,40] we were unable to show significant differences in migration, alignment, or positioning of the TKA components between the two groups. In contrast to our results, Parvizi et al. [9] noticed in a retrospective study about the risk factors for failure of TKA after previous HTO a high rate of femoral and tibial component loosening. They concluded that a reason for this may be that patients who had undergone a HTO are commonly younger and more obese. As a limitation they used matched control patients only for patients with bilateral TKA after a history of HTO, but did not use for all patients. Similar results were published by Kazakos et al. [31], and even though they recorded significant differences particularly in the radiographic evaluation after HTO, this did not compromise the clinical results.

Most data in literature as well as in the present study fail to detect considerable differences between subjects treated with TKA following HTO or primary TKA. In summary there seems to be a lack of randomised controlled trials. Well designed studies should investigate large numbers of subjects to generate higher quality of evidence. Furthermore, long-term results are needed to reach more solid conclusions.

## Conclusions

Although the results of clinical scores and radiological evaluation were quite similar in both groups patients after prior HTO showed significantly less range of motion. Surgeons should be aware that TKA is more challenging after HTO and was associated with more postoperative complications in the present study. However, satisfactory results with good survival can be achieved at mid-term follow-up.

## Competing interests

The authors declare that they have no competing interests.

## Authors' contributions

TE drafted the manuscript, and participated in its design and coordination; CB investigated, followed up and managed the patient, NT performed the statistical analysis; TJH, SFW, JS and CT participated in analysis and interpretation of data, MDS initiated the study, and participated in its design and coordination. All authors read and approved the final manuscript.

## Pre-publication history

The pre-publication history for this paper can be accessed here:

http://www.biomedcentral.com/1471-2474/11/207/prepub
